# Neurosarcoidosis: A Case Report

**DOI:** 10.7759/cureus.88041

**Published:** 2025-07-15

**Authors:** Alejandro Cardona, Sofia Castro, Naomi Córdoba, Valeria Cardona

**Affiliations:** 1 Pathology, Universidad Universidad Escuela de Ingeniería de Antioquia, Hospital Pablo Tobón Uribe, Medellín, COL; 2 School of Medicine, Universidad Universidad Escuela de Ingeniería de Antioquia, Hospital Pablo Tobón Uribe, Medellín, COL

**Keywords:** diagnosis, neurosarcoidosis, non caseating granulomas, report, sarcoidosis

## Abstract

This case report presents an atypical manifestation of sarcoidosis with neurological onset, characterized by paroxysmal seizure episodes accompanied by transient loss of consciousness in the absence of other clinical symptoms. Although sarcoidosis usually presents with multisystem involvement, neurologic manifestations are rare. This case emphasizes the importance of including neurosarcoidosis in the differential diagnosis of unexplained neurological events, particularly when granulomatous disease is suspected, and underscores the need to thoroughly exclude infectious and neoplastic causes.

## Introduction

Sarcoidosis is a systemic inflammatory disease characterized by the presence of non-caseating granulomas (organized aggregates of immune cells) in affected tissues. Although the exact cause remains unknown, the condition was first described by Besnier in 1889. It most commonly involves the lungs and thoracic lymph nodes, accounting for up to 90% of cases. However, in approximately 5-10% of patients, the central nervous system (CNS), which includes the brain and spinal cord, is affected, resulting in neurosarcoidosis, a less frequent but potentially serious form of the disease [1-4].

The most common presentations of neurosarcoidosis include intraparenchymal lesions and cranial neuropathies. Intraparenchymal lesions are abnormalities within the brain or spinal cord tissue itself and can lead to a wide variety of symptoms, depending on the area affected. Cranial neuropathies occur when the nerves that control vital functions such as vision, hearing, or facial movement are damaged, often causing facial weakness, blurred vision, or hearing loss. In addition, neurosarcoidosis can cause seizures, frequent headaches, or changes in mood, memory, and behavior, such as confusion, depression, or anxiety. Some people experience numbness, tingling, or weakness in the arms or legs, trouble walking, or problems with bladder and bowel control, especially when the spinal cord is involved. In rarer cases, it may present as cerebral vasculopathy (blood vessel inflammation in the brain) or encephalopathy (widespread brain dysfunction) [4-6].

When areas of the brain that regulate hormones are affected, symptoms like fatigue, excessive thirst, or hormonal imbalances can appear. Sleep issues and nerve pain in the limbs may also be present. Because neurosarcoidosis can affect many different parts of the nervous system, its symptoms are often diverse and sometimes difficult to recognize early. Diagnosis is challenging due to its clinical variability and overlap with other conditions. It is considered a diagnosis of exclusion, confirmed by a combination of clinical and radiological findings and the identification of non-caseating granulomas on biopsy [4-6].

This report presents a case of sarcoidosis with neurological onset, an uncommon manifestation that may go unrecognized. The main clinical forms, differential diagnoses, and key investigations necessary for its evaluation are discussed.

## Case presentation

This case report concerns a 44-year-old male patient with a personal history of arterial hypertension diagnosed in 2007, treated with losartan, smoking for one year in his youth, and a family history of a mother with rheumatoid arthritis. He denies other antecedents. He consulted for paroxysmal seizure events, characterized by episodes of disconnection, monophasy, global rigidity, and clonic movements.

**Figure 1:**
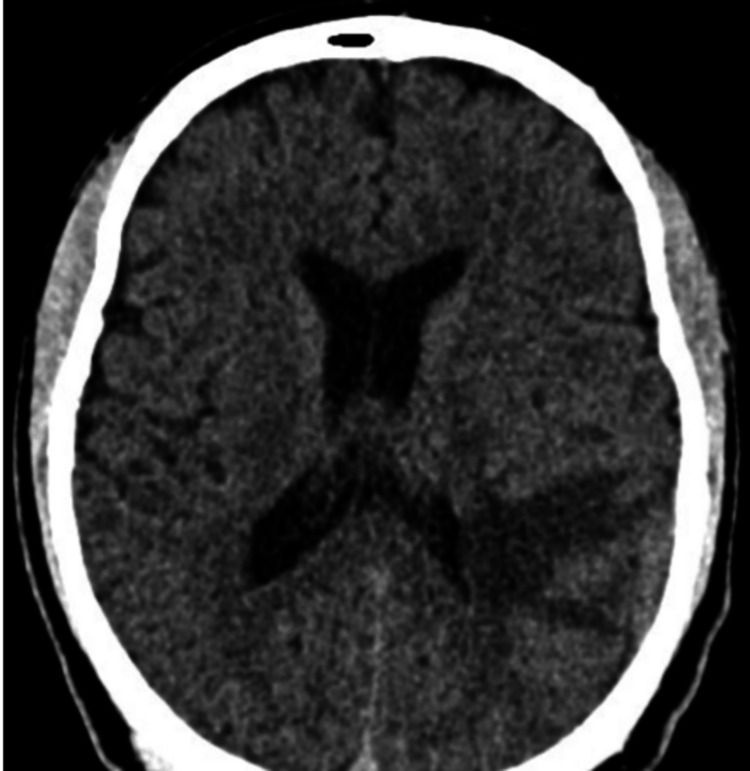
Magnetic resonance imaging Vasogenic edema of the left temporal lobe and inferior parietal lobe.

At the initial approach, the imaging and paraclinical search responsible for the neurological syndrome was determined, so an MRI was requested with evidence of a left hemispheric pachymeningeal lesion in the temporo-occipital region (Figure 1). 

In the paraclinical tests, human immunodeficiency virus (HIV) infection was ruled out, and cerebrospinal fluid analysis showed no inflammatory features. Venereal disease research laboratory (VDRL) and PCR results for *Mycobacterium tuberculosis* (MTB) were negative, as were aerobic cultures. Systemic studies such as electromyography/nerve conduction studies (ENA/NCS), antinuclear antibodies (ANAs), anti-cyclic citrullinated peptide antibody (antiCCP), and electrophoresis were performed with polyconic hypergammaglobulinemia, ruling out autoimmune and neoplastic causes; a very high level of IgG4 was evidenced, suggesting a possible IgG4 disease (Table 1).

**Table 1:** Laboratory results This table provides an organized summary of the patient’s laboratory results, categorized into five major clinical areas: inflammatory markers, hematology, immunology, autoimmune screening, microbiology, and CSF analysis. For each parameter, the table includes the measured result, the corresponding reference range, and a brief clinical interpretation. This format allows for a clear and concise evaluation of systemic inflammation, immune status, infectious screening, and central nervous system involvement. CRP: C-reactive protein; ESR: erythrocyte sedimentation rate; Hb: hemoglobin; MCV: mean corpuscular volume; IgM: immunoglobulin G; IgM: Immunoglobulin M; ANCA: anti-neutrophil cytoplasmic antibodies; ELISA: enzyme-linked immunosorbent assay; MPO: myeloperoxidase; PR3: proteinase 3; ENA: extractable nuclear antigens; HIV: human immunodeficiency virus; VDRL: venereal disease research laboratory; CSF: cerebrospinal fluid; WBC: white blood cells; RBC: red blood cells; ADA: adenosine deaminase; MTB: *Mycobacterium tuberculosis*

Category	Parameter	Result	Reference Range	Interpretation
Inflammatory Markers				
	CRP	2.7 mg/L	<5 mg/L	Normal
	ESR	109 mm/h	0–20 mm/h	Elevated
Hematology				
	Hb	15.4 g/dL	13.5–17.5 g/dL	Normal
	MCV	85 fL	80–100 fL	Normal
	Leukocytes	11,760/µL	4,000–11,000/µL	Mild leukocytosis
	Neutrophils	10,090/µL	2,000–7,500/µL	Neutrophilia
	Lymphocytes	988/µL	1,000–4,000/µL	Slightly low
	Platelets	240,000/µL	150,000–400,000/µL	Normal
Immunology				
	IgG	2818 mg/dL	700–1600 mg/dL	Elevated
	IgG4	8331 mg/dL	39.2–864 mg/dL	Significantly elevated – highly suggestive of IgG4-related disease
	IgG1	10,681 mg/dL	3,824–9,286 mg/dL	Mildly elevated
	IgG2	1623 mg/dL	1,000–1,500 mg/dL	Normal
	IgG3	1291 mg/dL	1,000–1,500 mg/dL	Normal
	IgM	93 mg/dL	40–230 mg/dL	Normal
	Serum protein electrophoresis	Polyclonal hypergammaglobulinemia	Normal pattern with no monoclonal bands	Suggests chronic inflammation or infection
Autoimmune Screening				
	ANCA ELISA (MPO/PR3)	Negative	Negative	Excludes ANCA-associated vasculitis
	ENA (RNP, Ro, La, SM)	Negative	Negative	Excludes common systemic autoimmune diseases
Microbiology tests				
	HIV	Non- reactive	Non-reactive	Excluded HIV infection.
	VDRL	Non-reactive	Non-reactive	No serologic evidence of syphilis
	Subgaleal secretion cultures	Aerobic cultures: negative	Negative	No aerobic bacterial growth; suggests no infection
	CSF WBC	0	0–5 cells/mm³	Normal; no signs of infection or inflammation
	CSF RBCs	10,000	0–10 cells/mm³	Elevated; may indicate traumatic tap or subarachnoid hemorrhage
	CSF protein	39.5 mg/dL	15–45 mg/dL	Normal
	CSF glucose	53mg/dL	40–70 mg/dL	Normal
	CSF ADA	0	<10 U/L	Normal; rules out tuberculous meningitis
	CSF flow cytometry	No evidence of hematolymphoid malignancy	N/A	No signs of leukemia or lymphoma in CSF
	CSF PCR for TB (MTB)	Negative	Negative	No evidence of tuberculosis
	CSF India ink stain	Negative	Negative	No evidence of cryptococcal infection

**Figure 2:**
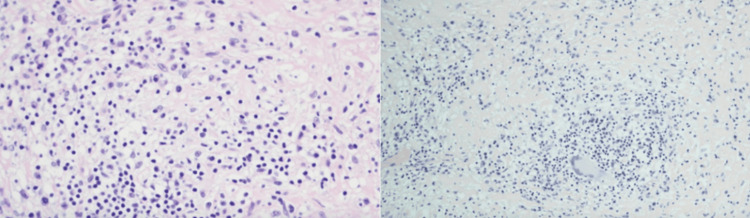
Biopsy of a mass located in the temporo-occipital lobe. H&E (hematoxylin and eosin) stained section revealed numerous epithelioid histiocytes forming granulomas. Scattered lymphocytes and sparse multinucleated giant cells are present. No evidence of central necrosis. Pathologic diagnosis of non-caseating granulomas.

Due to the failure to find the etiology of the lesion, it was decided to perform a resection of the extra-axial lesion, and a sample was taken for pathological analysis (Figure 2).

Empirical treatment was started with azathioprine 50mg once a day and prednisolone 50mg for the severe involvement of the patient, indicated for the two diagnostic suspicions, which were neurosarcoidosis or IgG4 disease.

The anatomopathological results showed non-caseating granulomas, with no evidence of infectious etiology. No storiform fibrosis was observed, and the IgG/IgG4 ratio was preserved, which ruled it out, so it was defined that the findings of the sample were compatible with neurosarcoidosis.

Postoperatively, he presented a superficial wound infection with subgaleal abscess, successfully treated with vancomycin and ceftriaxone. After resolution of the infection, immunosuppressive treatment was continued.

On clinical and imaging follow-up, the patient showed progressive improvement, with remission of symptoms. Currently, he is still being treated with azathioprine and has started a gradual reduction of corticosteroids.

## Discussion

Sarcoidosis is a granulomatous disease of unknown cause, secondary to an immune stimulus that generates a sustained activation of Th1 CD4 lymphocytes in the lung and other tissues, resulting in non-necrotizing granulomatous inflammation. The disease is mainly multisystemic and requires the presence of involvement in two or more organs for a specific diagnosis, considering that this is not the only pathology with the presence of granulomas, and it is necessary to rule out other etiologies when is suspected, as was the case with IgG4-related disease in this patient [7,8].

Neurosarcoidosis is most frequently reported in women and the African American population. Although this is not the most common affliction of sarcoidosis, its prevalence has been increasing due to advances in diagnostic methods and greater clinical awareness. It is estimated that neurosarcoidosis affects between 5% and 20% of patients with systemic sarcoidosis; however, some studies suggest that this percentage could be higher due to underdiagnosed cases [7,8]. In this case, IgG4-related disease was ruled out due to histopathological findings observed in the biopsy, which were consistent with sarcoidosis and did not show the typical features of IgG4-related disease [9].

Definitive diagnosis of neurosarcoidosis by biopsy is not always necessary in patients who already have evidence of systemic sarcoidosis. However, when neurological involvement is isolated and no involvement in other organs is identified, biopsy of the affected tissue in the central nervous system becomes the most conclusive diagnostic method. This should be done only after an exhaustive search for systemic disease that has not yielded clear results [10,11].

Among the possible infectious agents that are related to the highest incidence is *Propionibacterium acnes*, present in the lymph nodes of sarcoidosis patients. Other studies have shown the presence of a mycobacterial protein (*Mycobacterium tuberculosis *catalase-peroxidase) in the granulomas of some patients with sarcoidosis; this protein is resistant to degradation and may represent the persistent antigen in sarcoidosis [10-12] (Table 2). 

**Table 2:** Differential diagnoses of neurosarcoidosis Adapted from Badilla Nelson [11]. ANCA: anti-neutrophil cytoplasmic antibodies

Rheumatology	Hypersensitivity	Infection	Hematology	Other
Rheumatoid arthritis	Ag pneumonitis	Tuberculosis	Splenic lymphoma	Granulomatous hepatitis
Systemic lupus erythematosus	Drugs hypersensitivity	Aspergillosis	Castleman’s disease	Histocytosis
Necrotizing sarcoid granulomatosis		Histoplasmosis		Chronic granulomatosis
		Pneumocystis jirovecii		
		Syphilis		
Sjögren's syndrome		Cryptococcus		
Wegener’s granulomatosis				
Churg-Strauss				
IgG4				
Vasculitis ANCA				

The pathophysiology of sarcoidosis primarily involves alveolar macrophages, T cells, and T cell-associated antigens. Genetic loci related to antigen presentation, such as the HLA class II region and the butyrophilin-like 2 (BTNL2) gene, have been associated with disease susceptibility and specific phenotypic expressions. Antigen-presenting cells (APCs) utilize the HLA-CD4 complex to present an as-yet unidentified antigen to helper T cells. Studies have demonstrated that certain human leukocyte antigen (HLA) haplotypes, particularly HLA-DRB1 1101, are linked to an increased risk of developing sarcoidosis. The interaction between activated macrophages and CD4 T cells leads to the release of multiple cytokines, including interleukin-2 (IL-2) from T cells, and interferon-gamma (IFN-γ) and tumor necrosis factor (TNF) from macrophages. This cytokine-mediated response contributes to non-caseating granuloma formation [12,13].

Although granulomatous inflammation may resolve spontaneously or with treatment, approximately 20% of patients progress to a chronic form of the disease [12,13]. 

The clinical manifestations of neurosarcoidosis vary depending on the region of the central nervous system affected, however the cranial nerves are the most frequently affected (55%), followed by the meninges (12-40%), the brain parenchyma (20-45%), and the spinal cord (18-26.5%). Therefore, the most described symptoms include paresthesias, headache, confusion, seizures, cranial palsy, neuropathies, and paroxysmal seizure episodes, among others [13,14].

Currently, the most widely accepted diagnostic criteria classify neurosarcoidosis as definitive when there is direct confirmation of neural tissue, probable when there is evidence of neurological inflammation and systemic sarcoidosis, and possible when clinical presentation is typical, but no other criteria are met except the exclusion of other potential etiologies [14] (Table 3). Regarding treatment, glucocorticoids continue to be the first-line treatment for neurosarcoidosis. The decision to maintain monotherapy or add immunosuppressants depends on the dose, duration, and tolerability of the steroids. Recommendations for extrapulmonary disease are often similar, with adaptations depending on the location affected [15,16].

**Table 3:** Diagnostic criteria for neurosarcoidosis Adapted from Bradshaw et al. [16] CSF: cerebrospinal fluid; EMG: electromyography; NCS: nerve conduction studies

Diagnostic Category	Criteria
For all categories	The clinical presentation and diagnostic evaluation suggest neurosarcoidosis defined by the clinical manifestations and the findings of magnetic resonance imaging, CSF, and/or EMG/NCS typical of granulomatous inflammation of the nervous system and after the rigorous exclusion of other causes
Possible	There is no pathologic confirmation of granulomatous disease
Probable	There is pathological confirmation of systemic granulomatous disease compatible with sarcoidosis
Definitive	Type A. Extraneuronal sarcoidosis is evident; Type B. There is no evidence of extraneuronal sarcoidosis (isolated CNS sarcoidosis)

Clinical presentations of neurosarcoidosis vary widely, as previously mentioned. The intensity of treatment is guided by the specific clinical manifestation, with more aggressive therapy often required in severe or refractory cases. For example, facial paralysis can be treated with a few weeks of prednisone, with a low recurrence rate. On the other hand, in more severe cases such as bone medullary involvement, it is recommended to start from the beginning with second-line immunosuppressants such as methotrexate, azathioprine, mycophenolate mofetil, or cyclophosphamide; or even third-line drugs such as infliximab or adalimumab [16-18].

The main goal is to minimize neurological damage, so early diagnosis and prompt approach are critical. Although corticosteroids are effective in most cases with typical sarcoidosis, they are considered insufficient as the only therapy in neurological involvement. In severe cases of neurosarcoidosis, it is recommended to administer intravenous methylprednisolone pulses (1000 mg for 3-5 days), followed by oral prednisone at 1 mg/kg/day with gradual tapering [17,18].

The use of immunosuppressants as steroid-sparing agents is mandatory in neurosarcoidosis. Recommended doses include methotrexate (15-20 mg orally or subcutaneously per week), azathioprine (2 mg/kg/day), mycophenolate (1-1.5 g twice daily), hydroxychloroquine (200-400 mg/day), and leflunomide (10-20 mg/day, maximum 5 mg/kg). These medications take several months to reach clinical efficacy [18].

Cyclophosphamide, although effective and with a lower relapse rate, is currently less widely used due to its high toxicity, but it remains a valid option, especially in severe forms with vascular involvement. When there is toxicity or insufficient response, targeted therapies such as TNFα inhibitors are considered [16,18].

Infliximab (5-10 mg/kg) is the most studied and has been shown to be effective in combination with corticosteroids to treat refractory neurosarcoidosis, including severe forms affecting the meninges, brain, or spinal cord. Adalimumab is another option in this category [18].

Finally, the multidisciplinary approach is essential for the accurate diagnosis and comprehensive management of neurosarcoidosis, given the clinical complexity, the diversity of neurological manifestations, and the need to exclude multiple differential diagnoses that can simulate its presentation. This approach should include close collaboration between neurology, rheumatology, pulmonology, infectious diseases, radiology, immunology, and pathology, among other specialties, to systematically evaluate both nervous system involvement and possible systemic involvement.

The integration of clinical data, neuroimaging findings, cerebrospinal fluid analysis, serological studies, immunological tests, including, in the indicated cases, tissue biopsy, allows a more accurate and timely diagnosis to be established. Furthermore, this joint work is crucial to individualize treatment according to the location, severity, and progression of the disease, optimizing the use of glucocorticoids and immunosuppressants, and monitoring possible adverse effects and long-term complications. A coordinated approach also facilitates ongoing patient follow-up, which is essential given the chronic, recurrent, and potentially disabling nature of neurosarcoidosis. 

## Conclusions

This clinical case underscores the diagnostic and therapeutic complexity of neurosarcoidosis, especially when it presents as an isolated neurological syndrome without systemic involvement. The patient's initial manifestation with seizure episodes, absence of inflammatory findings in cerebrospinal fluid, and the detection of a temporo-occipital lesion on MRI illustrate the wide spectrum of possible presentations and the diagnostic challenges posed by this condition. It also emphasizes the value of immunohistochemistry and special stains such as CD3/CD68 for immune cell profiling and Ziehl-Neelsen to exclude mycobacterial infection.

Therapeutically, early initiation of corticosteroids combined with azathioprine allowed for symptom remission and radiological improvement, supporting current recommendations for immunosuppressive therapy in neurosarcoidosis. The favorable clinical evolution further highlights the importance of early recognition and timely intervention to prevent long-term neurological damage. Ultimately, this case exemplifies the need for high clinical suspicion, a structured diagnostic approach, and coordinated care among specialties. It also contributes to the limited literature on neurosarcoidosis with isolated CNS involvement, offering practical insights for clinicians facing similarly atypical presentations.
